# Vitiligo Associated with Melanoma in a Malagasy Woman

**DOI:** 10.1155/2019/7925785

**Published:** 2019-11-11

**Authors:** I. M. Ranaivo, F. A. Sendrasoa, M. Andrianarison, M. Razakanaivo, L. S. Ramarozatovo, F. Rafaramino, F. Rapelanoro Rabenja

**Affiliations:** ^1^Department of Dermatology, University Hospital Morafeno, Toamasina, Madagascar; ^2^Department of Dermatology, University Hospital Joseph Raseta Befelatanana, Antananarivo, Madagascar; ^3^Department of Oncology, University Hospital Joseph Ravoahangy Andrianavalona, Antananarivo, Madagascar

## Abstract

Malignant melanoma is the first fatal skin cancer. Vitiligo is a leukoderma or a multifactorial depigmentation acquired but especially of autoimmune origin. We report the first Malagasy case affected by both melanoma and Vitiligo. The appearance of Vitiligo during a melanoma could testify to an immunological response against melanocytes. Despite the association of melanoma and Vitiligo, the prognosis of melanoma is still fatal.

## 1. Introduction

Malignant melanoma is the most aggressive malignant tumor of skin cancer. Vitiligo is a multifactorial acquired leukoderma. The association of a malignant melanoma with various clinical forms of skin depigmentation is known in particular the association with a Vitiligo [1]. We report the first Malagasy case of melanoma associated with and Vitiligo.

## 2. Case Presentation

**Figure 1:**
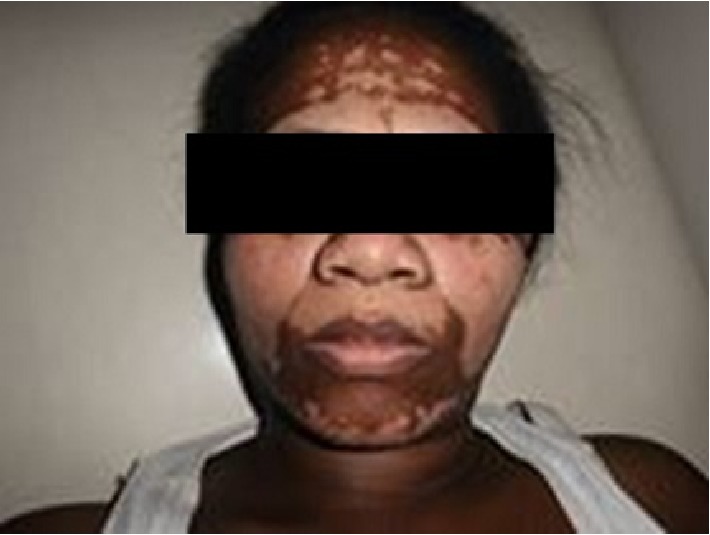
Facial achromic lesions (archive of the Department of Dermatology, Joseph Raseta Befelatanana University Hospital, Antananarivo, Madagascar).

**Figure 2:**
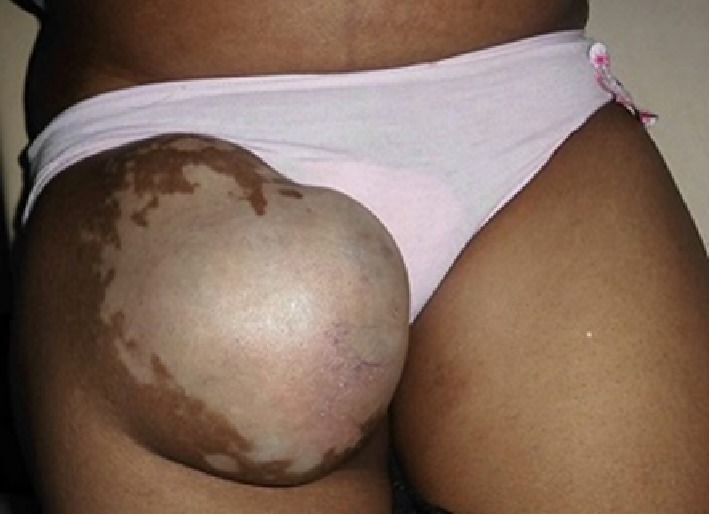
Achromic lesion with right inguinal swelling (archive of the Department of Dermatology, Joseph Raseta Befelatanana University Hospital, Antananarivo, Madagascar).

**Figure 3:**
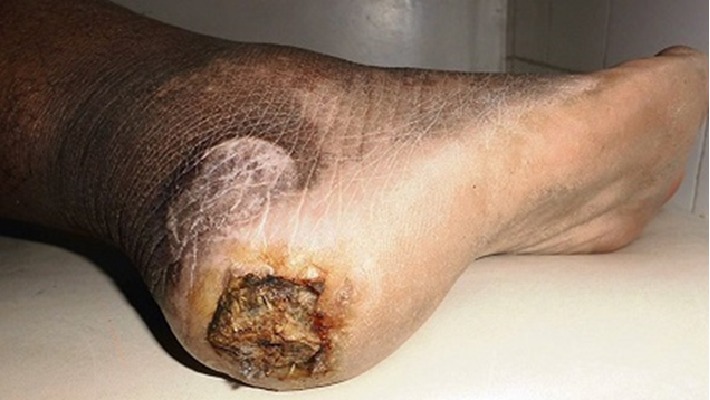
Ulcerous lesion of the right heel (archive of the Department of Dermatology, Joseph Raseta Befelatanana University Hospital, Antananarivo, Madagascar).

A 44-year-old woman, farmer, presented asymptomatic skin depigmentations associated with a non-painful right inguinal swelling evolving for 6 months. Two years ago, she had an excision of a tumor lesion pigmented in the pressure zone of right heel, but no histology screen was performed. The pigmented lesion of the heel recurred one year later, motivating a second surgical resection of which histology analysis shown specific signs of lentiginous melanoma, Breslow >4 mm, Clark IV, with ulceration and positive margin. She had no family history of melanoma or Vitiligo. Physical examination show that the patient was in good general condition. She had multiple achromic patches varying in size from a few millimeters to 20 cm in diameter, in the face (Figure 1), chest, arms, thighs, right inguinal swelling, and backs of the feet. She had also a hard right inguinal lymphadenopathy 15 cm in diameter adhering to the deep plane (Figure 2). On the right heel, she had a pigmented ulcero-crusty lesion of 4 cm in diameter (Figure 3).

The rest of the physical examination was normal. The extension assessment found liver metastases and necrotic lymphadenopathy on the right iliac chain. Then, a diagnosis of acral lentiginous melanoma, Stage IV according to American Joint Committee on Cancer 2009 (AJCC) associated with recent generalized Vitiligo was made. She received palliative treatment. Unfortunately she died 2 months later.

## 3. Discussion

In Madagascar skin cancers account for 9% of cancers and melanomas account for 23% of malignant skin tumors [2]. Vitiligo is an acquired leukoderma due to destruction or loss of melanocytes. It is probably a multifactorial disease: genetic and environmental factors as well as autoimmune and auto-inflammatory mechanisms have been advanced [3–5]. Various pathogenetic mechanisms have been suggested, most evidence supports an autoimmune basis for the Vitiligo. Different genome-wide association studies of generalized Vitiligo have been reported, identifying a total of 17 confirmed generalized Vitiligo susceptibility loci. Almost all of these genes encode immunoregulatory proteins. In melanocytes HLA-A∗02:01 presents the major Vitiligo autoimmune antigen, TYR (encoding tyrosinase), which in turn, activates and recruits anti-melanocytes auto-reactive cytotoxic T lymphocytes to the skin within the target and destroy melanocytes. Moreover, the biological interaction between these generalized Vitiligo susceptibility genes, HLA-A and TYR, points to show inverse relationship between susceptibility to generalized Vitiligo versus malignant melanoma, suggesting that generalized Vitiligo may result, in part, from dysregulation of normal processes of immune surveillance against melanoma. Unfortunately, we could not do genetic study for our patient to confirm this hypothesis [6, 7].

Vitiligo-like lesions may appear during primary or metastatic melanoma [8]. It affects 3% to 6% of melanoma cases. Leukoderma can precede, from a few months to several years, the appearance of melanoma [1, 9]. Or it can appear on primary or metastatic melanoma spontaneously or during melanoma treatment. The development of Vitiligo during melanoma maybe the result of an immunological response against melanocytes. Humoral and cellular immunity is involved [9]. In particular, the antityrosinase antibodies and the CD8 + oligoclonal in vitro T lymphocytes were involved in the destruction of normal melanocytes during the immune response to melanoma antigens [10, 11]. During immunotherapy by pembrolizumab in patients with metastatic melanoma, the appearance of Vitiligo demonstrates a good therapeutic response [12]. In common Vitiligo, there is a greater history of autoimmune disease and family history of Vitiligo compared to patients with Vitiligo-like lesions during melanoma. Achromic lesions can localize around cutaneous and distant metastases. They are often generalized bilateral, symmetrical and without Koebner phenomenon in case of Vitiligo associated with melanoma [10, 13]. For our patient she had a generalized Vitiligo with achromic lesion next to lymph node metastasis. Although the presence of Vitiligo indicates a strong immune response against melanoma, our patient died quickly. In Madagascar the management of melanoma especially in the metastatic stage is disappointing. The treatment is expensive, we do not have molecular biology yet. Immunotherapy and targeted therapy are not yet available.

Table 1 shows some cases of Vitiligo associated with melanoma reported in the literature.

## 4. Conclusion

Our patient's case illustrates an occurrence of concomitant Vitiligo and melanoma, presumably the first reported case in Madagascar. Although the melanoma-Vitiligo association is an indication of an immunological response against normal melanocytes and melanoma antigens, the prognosis of melanoma is still fatal. In Madagascar the treatment of melanoma is difficult especially in case of metastatic forms. More attention should be given to the examination of melanocyte lesions in adult patients in order to detect melanoma at the onset stage. Malignant melanoma can be overlooked by patients, as in the case of our patient who was concerned about the presence of cosmetic disfiguring achromic lesions.

## Figures and Tables

**Table 1:** Comparative analysis of some cases of melanoma associated with Vitiligo reported in the literature.

	Redondo and Del Olmo [14]	Alonso-Castro et al. [15]		Flores-Terry et al. [16]	Wang et al. [10]	Our case
Age	65	63	35	44	67	44
Gender	Woman	Woman	Woman	Woman	Man	Woman
Primary melanoma	Right leg	Not precised	Not precised	Left outer ear	Left sole	Rigth sole
Metastasis	Lymph nodes metastase	Pulmonary, bone, and axillary lymph nodes metastases	Lymph nodes metastases	Lymph nodes and pulmonary metastases	Lymph nodes	Lymph nodes and liver metastases
Vitiligo locations	Face and trunk	Face and neck	Face and neck	Face, scalp, trunk, and limbs	Face, trunk and limbs	Face, arms, trunk, and inguinal
Treatment	Surgical resection and chemotherapy	Target therapy vemurafenib	Target therapy vemurafenib	Target therapy dabrafenib	Chemotherapy	Palliative treatement
Delays of appearance of Vitiligo after the melanoma's discovery	2 months after the discovery of melanoma	After 2 weeks of treatment	After 8 weeks of treatment	After 2 months of treatment	4 months before the discovery of melanoma	2 years after the discovery of melanoma
Evolution	Death	Partial response	Complete response	Clinical regression	Death	Death

## References

[1] 
Baghad B., Belanouane S., Elfatoiki F., Baline Z., Hali K. F., Chiheb S. (2018). Association mélanome et Vitiligo: quelle relation?. *Annales de Dermatologie et de Vénéréologie*.

[2] Raharisolo Vololonantenaina C., Pécarrère J. L., Roux J. F. Le cancer à Madagascar. Expérience de l’Institut Pasteur de Madagascar de début septembre 1992 à fin juin 1996. http://www.pathexo.fr/documents/articles-bull/T91-1-MR96-120.pdf.

[3] Ezzedine K., Eleftheriadou V., Whitton M., van Geel N. (2015). Vitiligo. *The Lancet*.

[4] Rapelanoro Rabenja F., Randrianasolo F. M. P., Ramarozatovo L. S., Ratrimoarivony C. (2005). Therapeutic observation of Vitiligo. *International Journal of Dermatology*.

[5] Sendrasoa F. A., Ranaivo I. M., Sata M. (2019). Treatment responses in patients with Vitiligo to very potent topical corticosteroids combined with vitaminotherapy in Madagascar. *International Journal of Dermatology*.

[6] Jin Y., Hayashi M., Fain P. (2015). Major association of Vitiligo with HLA-A∗ 02: 01 in Japanese. *Pigment Cell & Melanoma Research*.

[7] Spritz R. A. (2010). The genetics of generalized Vitiligo: autoimmune pathways and an inverse relationship with malignant melanoma. *Genome Medicine*.

[8] Saleem M. D., Oussedik E., Schoch J. J., Berger A. C., Picardo M. (2019). Acquired disorders with depigmentation: a systematic approach to vitiliginoid conditions. *Journal of the American Academy of Dermatology*.

[9] González R., Torres-López E. (2014). Immunological basis of melanoma-associated Vitiligo-like depigmentation. *Actas Dermo-Sifiliográficas (English Edition)*.

[10] Wang J. R., Yu K. J., Juan W. H., Yang C. H. (2009). Metastatic malignant melanoma associated with Vitiligo-like depigmentation. *Clinical and Experimental Dermatology*.

[11] Becker J. C., Guldberg P., Zeuthen J., Bröcker E.-B., thor Straten P. (1999). Accumulation of identical T cells in melanoma and Vitiligo-like leukoderma. *Journal of Investigative Dermatology*.

[12] Hua C., Boussermart L., Mateus C. (2016). Association of Vitiligo with tumor response in patient with melanoma treated with pembrolizumab. *JAMA Dermatology*.

[13] Lommerts J. E., Teulings H.-E., Ezzedine K. (2016). Melanoma-associated leukoderma and Vitiligo cannot be differentiated based on blinded assessment by experts in the field. *Journal of the American Academy of Dermatology*.

[14] Redondo P., Del Olmo J. (2008). Vitiligo and cutaneous melanoma. *New England Journal of Medicine*.

[15] Alonso-Castro L., Rıos-Buceta L., Vano-Galvan S., Moreno C., Soria-Rivas A., Jaén P. (2013). Vitiligo in 2 patients receiving vemurafenib formetastatic melanoma. *Journal of the American Academy of Dermatology*.

[16] Flores-Terry M. A., Garrido-Martın A., Sanchez-Mateos L. S., Sánchez-Caminero M. P., García-Arpa M., Cortina-de la Calle M. P. (2016). A case of Vitiligo-like depigmentation induced by dabrafenib in patients with metastatic melanoma. *Journal of the American Academy of Dermatology*.

